# Application of advanced causal analyses to identify processes governing secondary organic aerosols

**DOI:** 10.1038/s41598-024-59887-7

**Published:** 2024-05-10

**Authors:** S. Sinha, H. Sharma, M. Shrivastava

**Affiliations:** The Pacific Northwest National Laboratory, Richland, WA 99354 USA

**Keywords:** Climate sciences, Atmospheric science, Atmospheric chemistry

## Abstract

Understanding how different physical and chemical atmospheric processes affect the formation of fine particles has been a persistent challenge. Inferring causal relations between the various measured features affecting the formation of secondary organic aerosol (SOA) particles is complicated since correlations between variables do not necessarily imply causality. Here, we apply a state-of-the-art information transfer measure coupled with the Koopman operator framework to infer causal relations between isoprene epoxydiol SOA (IEPOX-SOA) and different chemistry and meteorological variables derived from detailed regional model predictions over the Amazon rainforest. IEPOX-SOA represents one of the most complex SOA formation pathways and is formed by the interactions between natural biogenic isoprene emissions and anthropogenic emissions affecting sulfate, acidity and particle water. Since the regional model captures the known relations of IEPOX-SOA with different chemistry and meteorological features, their simulated time series implicitly include their causal relations. We show that our causal model successfully infers the known major causal relations between total particle phase 2-methyl tetrols (the dominant component of IEPOX-SOA over the Amazon) and input features. We provide the first proof of concept that the application of our causal model better identifies causal relations compared to correlation and random forest analyses performed over the same dataset. Our work has tremendous implications, as our methodology of causal discovery could be used to identify unknown processes and features affecting fine particles and atmospheric chemistry in the Earth’s atmosphere.

## Introduction

Secondary organic aerosols (SOA) are key components of fine particles in the atmosphere, which scatter and absorb incoming solar radiation, and seed clouds by acting as cloud condensation nuclei (CCN), and these interactions between aerosols, clouds and radiation constitute one of the largest uncertainties of climate forcing^1,2^. SOA are complex systems since they represent thousands of organic species that dynamically interact in the atmosphere^2^.

Analyzing causal relationships between SOA and other chemistry and meteorological variables could help identify additional unknown but important processes in fine particle formation in the atmosphere. To this end, an analysis of the time series of related variables through some of the latest data discovery and information transfer tools holds a lot of promise^3–5^ and to the best of our knowledge, these tools have not been explored in the context of SOA formation. While correlation analyses of field measurements of SOA and related chemistry and meteorological variables can be useful in understanding related features, these correlations do not necessarily imply causality. Before applying causal tools to field measurements, it is important to test causal tools against the already well-known mechanistic relationships of SOA formation pathways and their governing variables. Field datasets could have several confounding factors and noise and are sparse in space and time. Detailed regional model predictions are a good alternative to test causal tools since they provide detailed representations of processes that are continuous in space and time over given domains of interest. Here, we test causal relationships predicted by the latest information transfer machine learning tools that we apply to IEPOX-SOA concentrations and associated governing variables, which have been simulated within a three-dimensional regional model, the Weather Research and Forecasting Model Coupled to Chemistry (WRF-Chem)^6^. WRF-Chem simulates the feedback between aerosol-radiation-cloud-chemistry interactions and meteorology. We conduct causal analyses using only the time series of the WRF-Chem outputs to test if the causal and information transfer results are consistent with our physical and chemical understanding of IEPOX-SOA formation. Since 2-methyltetrols and their oligomers in the particle phase were predicted to be the dominant components of IEPOX-SOA over the Amazon rainforest^6^, here we focus our analyses on particle phase 2-methyltetrols.

Causal analysis identifies cause-and-effect relationships and thus gives insight into any process. Though the understanding of the causal structure has been an important question in science and engineering, there is no unified working definition of causality and it depends on the underlying system being analyzed. Among the various definitions of causality, the most commonly used is Granger causality^7,8^, which was first proposed in 1969 in the context of the analysis of econometric data. On the other hand, using concepts from Shannon’s information theory^9^, Massey and Kramer defined *directed information*^10^ and Schreiber defined *transfer entropy*^11^ as measures of causality. Recently, Machine Learning (ML)-based techniques like Random Forest (RF)^12^ have been used to identify the important features in a multivariate data set.

However, it has been since shown that in the context of dynamical systems, these measures fail to capture the true causal structure^3^. To this end, in Refs.^3,4^ the authors defined a new notion of causality, called *information transfer*, which quantifies the cause-effect relationships between the various states (subspaces in general) of a dynamical system. It was shown in^3,4^ that the information transfer measure can capture the true causal structure of a dynamical system, and it can also be used to characterize influence in a dynamical system^13,14^. Furthermore, in^5,15^ the authors provided data-driven techniques for computing the information transfer measure from time-series data. In this work, we leverage the data-driven computation framework for inferring causality in atmospheric chemistry and IEPOX-SOA formation. We describe this algorithm in more detail in the Supplementary Material and provide two examples to illustrate how information transfer measure can be used to infer causal links for a chemical process (See Supplementary Figs. S4 and S5).

In this paper, we assess the utility of information transfer approaches to predict the known causal relationships between key features governing 2-methyltetrol particle evolution, as simulated by the WRF-Chem model. We also compare the information transfer analyses with simple correlation analyses and demonstrate how confounding factors might prevent the interpretation of correlation to infer causality. Furthermore, we also used random forest (RF) analyses to identify important features that affect 2-methyltetrols and show via examples that though RF captures key feature importance, it does not necessarily imply causality. RF feature importance represents the association between a given variable and the predicted outcome. This understanding of associations provides valuable insights about which variables govern the model’s predictions, but it doesn’t establish cause-and-effect relationships. To understand causality, we need to go beyond a mere understanding of feature association. Our information transfer approach shows great promise in identifying the key variables affecting IEPOX-SOA formation and also successfully identifying the causal structure amongst the variables. We view this as the first application and success of causality approaches in atmospheric chemistry and SOA formation. In the future, this approach could likely be applied to well-characterized field and laboratory measurements.

## Information transfer and causality in dynamical systems

Identifying causal relationships among different interacting variables has been an active area of research since the days of Aristotle. Measures like correlation and mutual information capture the interaction between two variables, but these measures are symmetric, and as such, they do not contain any information regarding which variable is the cause and which is the effect. To this end, depending on the application, various definitions, like Granger causality^7,8^, transfer entropy^11^, bidirectional information^16^ etc., have been proposed. However, these measures capture a sort of statistical dependence, and they all fail to infer the causal structure of a dynamical system. This is because it does not account for the underlying dynamical nature of the evolving states. Hence, a measure that can identify the true causal structure of a dynamical system should inherently take into account the dynamical nature of the system. To this end, a new quantitative measure of causality, called *information transfer*, has been recently developed^3,4^ and has been shown to capture the correct causal structure of a dynamical system. In particular, the main advantages of the information transfer measure are the following:

- Unlike correlation, it quantifies the influence of one dynamic variable on any other dynamic variable.
- It can differentiate between direct and indirect influence.
- It can account for confounding variable effect.

The intuition behind the definition of information transfer from a dynamic state *x* to another dynamic state *y* is the fact that the total entropy of a dynamic variable *y* can be decomposed as the sum of the entropy of *y* when a given dynamic variable *x* is not present in the system and the entropy transferred from *x* to *y*. The information transfer from *x* to *y* depicts how the evolution of *x* affects the evolution of *y*; that is, it gives a quantitative measurement of the influence of *x* on *y* and is thus inherently asymmetric. For details of the mathematical formulation of the concept of information transfer, we refer the reader to Refs.^3,4^ and the Supplementary Material.

In general, the value of the information transfer measure from a state *x* to another state *y* can change with time during transient dynamics. However, stable dynamical systems always admit a stable steady state distribution^17^ and hence the system entropies and information transfer values do not change with time at steady state. In this paper, since the underlying system of data from WRF-Chem predictions is stable, we consider the steady-state information transfer values for all subsequent analyses.

Note that the computation of the information transfer measure requires knowledge of the evolution of entropy under the flow of the underlying dynamical system. However, there are no general closed-form expressions for the evolution of entropy for general nonlinear systems. To circumvent this problem, we use the Koopman operator framework^17–19^ for computing the information transfer from time-series data.

**Figure 1:**
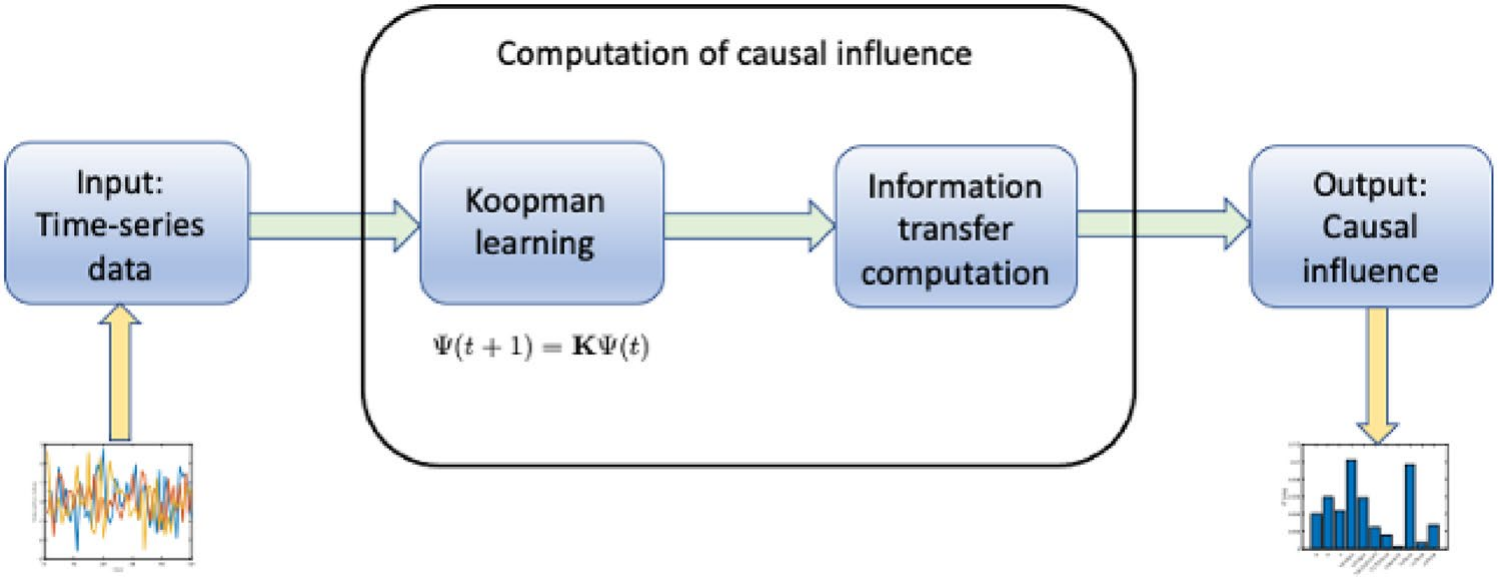
Computation of information transfer from time-series data: We have an unknown dynamical system from which time-series measurements $[x_1,\cdots , x_M]\in {\mathbb {R}}^{n\times M}$ of the states are obtained. The obtained time-series data is lifted to a space of functions using the Koopman observables $\Psi (x)$, where the dynamics is represented by the linear Koopman operator. Thus, Koopman learning identifies a linear model^17,18^ of the underlying dynamical system, and the linear model is used to compute the relevant conditional entropies, which in turn are used to compute the information transfer measure.

The Koopman operator is a linear operator and gives an exact linear representation of any general nonlinear dynamical system. However, the trade-off lies in the fact that the Koopman operator is typically infinite-dimensional, and hence, in practice, one needs to compute a finite-dimensional approximation of the Koopman operator^20^. The learnt Koopman operator is a linear representation of the underlying dynamical system, which is described further in the Supplementary Material (see Fig. 1 and the Supplementary Material). Note that the Koopman operator framework can be thought of as a nonlinear change of coordinates, which changes the nonlinear evolution of the dynamical system (nonlinear on Euclidean space) to a linear evolution on the space of functions. In particular, the time-series data is lifted to a space of functions using the observable functions ($\Psi$ in Fig. 1), where the evolution is always linear (in particular, it is given by a linear combination of the non-linear observable functions), and this linear Koopman model is valid for the entire state space. Once a linear system is obtained from the time-series data, one can use the closed-form expressions for conditional entropy for linear systems to compute the information transfers. For details of the computation of information transfer from time series data, see^5,15^ and the Supplementary Material.

## Some properties of information transfer measure

The main difference between any measure of causality and correlation is the fact that the measure of causality should be asymmetric. It is this asymmetry that captures the direction of influence between two variables, which measures like correlation or mutual information can not capture. Furthermore, a good measure of causality should also be able to capture the effect of confounding variables.

### Causation v/s correlation

Correlation or dependence is any statistical relationship, whether causal or not, between two random variables and inherently it is symmetric. Correlations are useful because they can indicate a predictive relationship that can be exploited in practice. However, it cannot differentiate between the cause and the effect. Similarly, mutual information between two random variables does not capture the causal relationship between the random variables.

**Figure 2:**
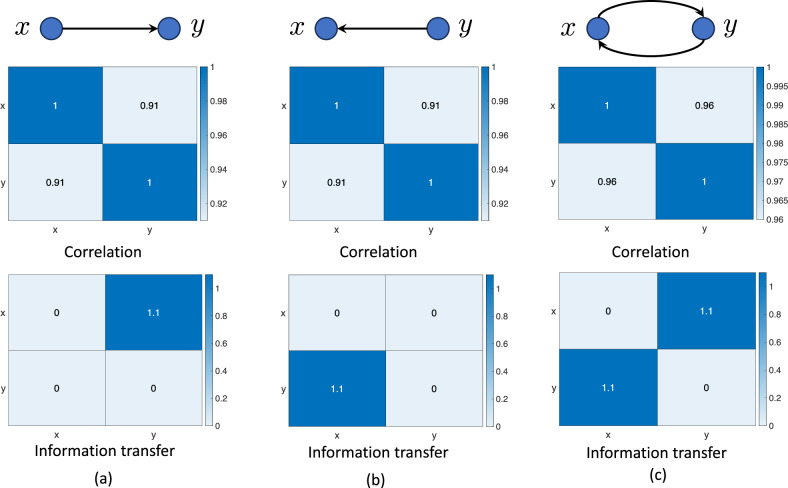
Consider three dynamical systems with two states *x* and *y*. In the first system (**a**), the state *x* affects the dynamics of the state *y*, while *y* does not affect *x*. In the second system (**b**), the state *y* affects the dynamics of the state *x*, while *x* has no effect on the dynamics of *y*. In the third system (**c**), both states affect each other. However, in all three systems, there is a non-zero correlation between the variables *x* and *y*, whereas the causal structure is different in the three systems.

To illustrate the difference between correlation and causation and how information transfer measure can differentiate between correlation and causation, consider three different dynamical systems as shown in Fig. 2. For simplicity, we consider two-dimensional linear dynamical systems with states *x* and *y*. In the first system, the state *x* affects the dynamics of *y* and thus *x* is the *cause* and *y* is the *effect*. In the second case, *y* is the cause and *x* is the effect and in the third system, both *x* and *y* affect each other. As can be seen from the correlation values, for all the systems, the states are highly correlated. However, information transfer from *y* to *x* is zero for the first system (Fig. 2a), while the information flow from *x* to *y* is non-zero, thus identifying the correct causal structure. Similar observations can be made for the other systems as well. Thus, the information transfer measure unearths more information about a dynamical system than mere correlation.

### Information transfer and confounding variables

As mentioned earlier, one of the drawbacks of measures of causality like Granger causality is that it fails to capture the causal structure of a dynamical system. Moreover, it cannot account for confounding variables as well. In this subsection, we illustrate, via a simple example, how the information transfer measure can account for confounding variables as well. Consider a linear system whose equations of motion are

1 $$\begin{aligned} \begin{pmatrix} x_1(t+1)\\ x_2(t+1)\\ x_3(t+1)\\ x_4(t+1)\\ x_5(t+1) \end{pmatrix} = \begin{pmatrix} 0 &{} 0 &{} 0 &{} 0 &{} 0\\ 1 &{} 0 &{} 0 &{} 0 &{} 0\\ 0 &{} 1 &{} 0 &{} 0 &{} 0\\ 0 &{} 1 &{} 0 &{} 0 &{} 0\\ 0 &{} 0 &{} 0 &{} 1 &{} 0 \end{pmatrix} \begin{pmatrix} x_1(t)\\ x_2(t)\\ x_3(t)\\ x_4(t)\\ x_5(t) \end{pmatrix} + \xi (t), \end{aligned}$$

where $\xi (t)\in {\mathbb {R}}^5$ is an independent and identically distributed (i.i.d.) Gaussian noise with zero mean and unit variance.

**Figure 3:**
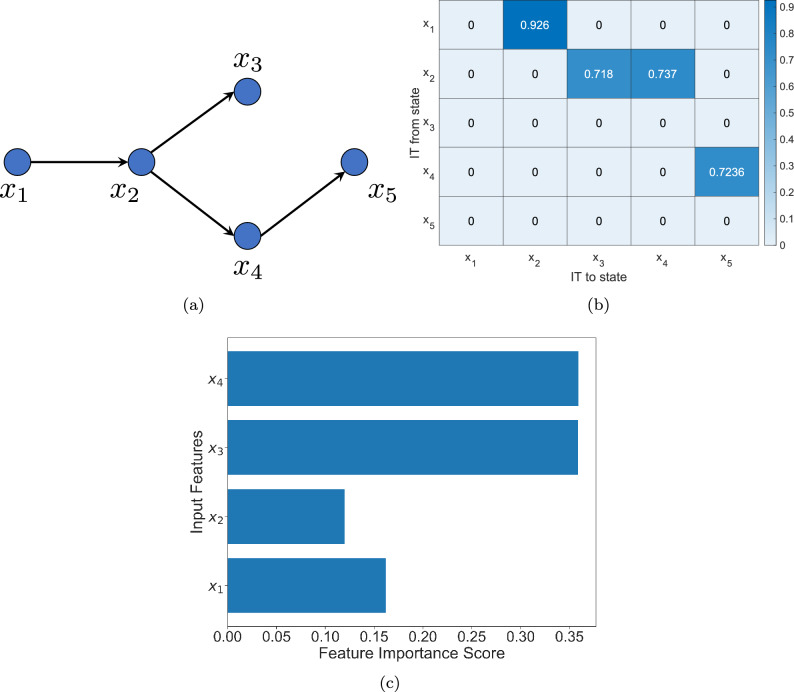
(**a**) The causal graph of the 5-dimensional linear system, as obtained from the system equations (1). (**b**) The information transfer between the states. (**c**) Important features identified by Random Forest.

From Eq. (1), it can be seen that the $x_2$ dynamics is affected by $x_1$, the $x_3$ dynamics is affected by $x_2$ and so on and so forth. The causal structure derived from equation (1) is shown in Fig. 3a. Here, $x_3$ and $x_4$ are confounding variables and though there is no causal link between them, the correlation between them is non-zero. In Fig. 3b we show the information transfer values between all the states and it can be seen that the information transfer from any state $x_i$ to $x_j$ is non-zero if and only if there is a causal link (directed edge) from $x_i$ to $x_j$. Thus, information transfer can account for confounding variables as well. For theoretical proofs as to why this is so, we refer the reader to^3,4^. We also compare the information transfer measure with Random Forest (RF), which identifies and quantifies feature importance.

The Random Forest (RF)^12^ technique is widely recognized as one of the most favored ensemble model-based approaches in the field of machine learning and is a non-parametric algorithm that can capture complex relationships between features and the target variable without making any assumptions about linearity. This algorithm combines multiple decision trees to generate predictions and has gained popularity due to its high accuracy, robustness, and interpretability, making it a preferred choice for both classification and regression tasks. The Random Forest algorithm begins by creating a collection of decision trees. Each tree is constructed using a random subset of the training data and a random subset of the available features. This randomness in the selection of both the data and features ensures that each tree produces a unique outcome. The strength of the RF lies in its ability to aggregate the predictions of multiple decision trees. By combining the outputs of these individual trees, the algorithm is able to generate a final prediction that is more accurate and reliable than that of any single tree. Additionally, the use of random subsets helps to reduce overfitting, making the algorithm more robust and less prone to errors. Furthermore, the Random Forest algorithm provides interpretability, allowing users to understand the factors that contribute to the final prediction. By examining the importance of different features across the ensemble of trees, one can gain insights into the underlying patterns and relationships within the data. The feature importance calculated by RF is not based on correlation because RF operates based on the principle of impurity reduction rather than correlation analysis. On the other hand, correlation analysis assumes a linear relationship between variables, which may not always hold true in real-world datasets. It evaluates the importance of features based on their ability to split the data and reduce impurity, which is a more robust and flexible approach. The RF approach computes the mean decrease in impurity (or Gini importance) and aims to quantify the extent to which a feature reduces the impurity or uncertainty in the predictions made by the decision trees within the random forest. Additional details about the impurity-based approach can be seen in ref. ^21^.

Though RF can identify the important features for predicting one variable, it does not identify the causal structure, especially in the presence of confounding variables. To illustrate this, we consider the dynamical system (1) and compare the information transfer to the state $x_5$ and the important features identified by RF. The results are presented in Fig. 3 and it can be seen from Fig. 3b that only $x_4$ has non-zero information transfer (and hence non-zero influence) to $x_5$, whereas RF identifies all the other states as *important* for predicting $x_5$ (Fig. 3c). Hence, we conclude that the information transfer measure is far superior to RF for inferring causality.

### Causation in presence of unknown state(s)

**Figure 4:**
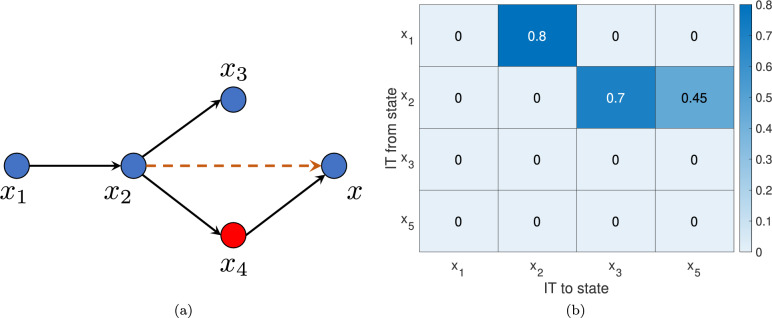
(**a**) A five dimensional system, such that we have time-series data of $x_1$, $x_2$, $x_3$ and $x_5$. In this case, any learning algorithm (including any ML/DL method) won’t know that there are 5 states and the learned model will be such that it describes the obtained 4-dimensional data (ML is ultimately curve-fitting). Thus, any causal analysis will give the causal structure of this 4-dimensional system. (**b**) The information transfer between the states.

The information transfer framework identifies the direct causal relationships between the states of a dynamical system and this measure can be computed from models or from time-series data of the states of the system. However, in many real world examples, often it might not possible to obtain the measurements of all the states, especially measurements of more complicated features. Additionally, one might not know all dynamic states and independent features affecting a given variables of interest in the system. For example, if we have time-series measurements of *m* states of a system and it is not known whether there are exactly *m* states or more than *m* states. It might happen that some state *x* directly affects state *y*, which in turn directly affects state *z*. But that there is no direct causal link from *x* to *z*. Suppose we don’t have the measurement of *y*. In this case the information transfer from *x* to *z* will be non-zero. For example, in a linear system (1), consider that we don’t have the measurement of given variable $x_4$ and we do not know that the system has five states (see Fig. 4). In this case, any learning algorithm (no matter how *advanced*) will output a 4-dimensional model which explains the obtained time-series data (because the algorithm does not know that the system has five states instead of four). Thus, the causal structure obtained will not be the exact causal structure. However, in this case, the information transfer measure should capture a *direct* link from $x_2$ to $x_5$ (since $x_4$ is not known in the system) and from Fig. 4b we see that this is indeed the case. Moreover, we also find that apart from the causal link from $x_2$ to $x_5$, the information transfer measure does capture the other causal links properly. Thus, in the presence of unknown unmeasured variables, the information transfer measure captures the indirect causal dependencies via the unmeasured variable (in this case $x_4$).

## Results

The goal of this work is to identify the variables that causally affect total tetrol (the major component of IEPOX-SOA), which is the sum of particle-phase tetrol and tetrol oligomer. Since the causal effects on total tetrol are coded in the aqueous chemistry module in WRF-Chem, based on our domain knowledge from aqueous chemistry, we use the key dynamic variables affecting IEPOX-SOA, as listed in Table 1. The task of the causal model is to identify the causal influence of these dynamic variables on IEPOX-SOA.

**Table 1:** Dynamic variables Considered.

Full name	Acronym
Temperature	tk
Relative humidity	rh
Ambient pressure	p
Isoprene epoxydiol (IEPOX) gas	iepoxgas
2-methyltetrol gas	tetrolgas
Glass transition temperature of organic aerosol	tglasscoat
Total organic aerosol	OA
Particle water	Water
Particle sulfate	$SO_4$
Particle nitrate	$NO_3$
Particle ammonium	$NH_4$
Particle tetrol oligomer	tetolig
Particle tetrol	tetrol

We analyze the causal effects on tetrols at two different altitude levels, namely near the earth’s surface (0–500 m) and high in the atmosphere (10–12 km). Near the Earth’s surface, both aerosol/cloud chemistry and surface chemistry on leaves and soils might contribute to IEPOX-SOA formation; however, at high altitudes in the upper troposphere (10-12 km altitudes), aerosol/cloud chemistry is shut off due to a lack of aerosol and cloud liquid water at extremely cold temperatures ($\approx$225 K) at those altitudes^6^. In the upper troposphere, tetrol gases emitted near the surface and transported by clouds from the surface were suggested to mainly contribute to IEPOX-SOA^6^. Due to differences in processes affecting IEPOX-SOA at high altitudes compared to the surface, we selected these two altitude ranges as test cases for evaluating our causal approaches. The causal relations between IEPOX-SOA and other chemistry and meteorological features included in WRF-Chem are our ground truth for this study, against which we tested three different approaches: correlation, RF and information transfer.

**Figure 5:**
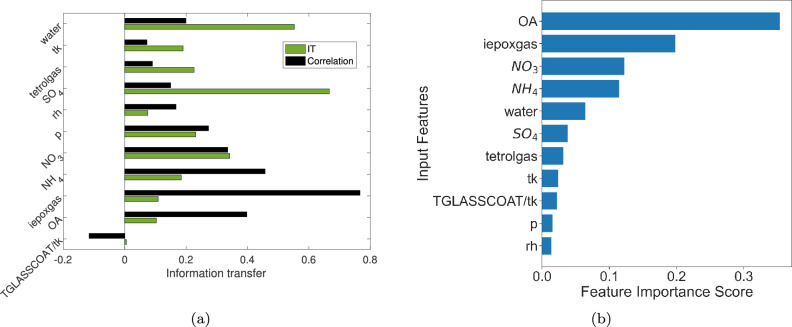
(**a**) Information transfer to total tetrol and correlation of total tetrol with other variables for altitude 0-500m. (**b**) Feature importance identified by Random Forest for total tetrol for altitude 0-500m.

**Figure 6:**
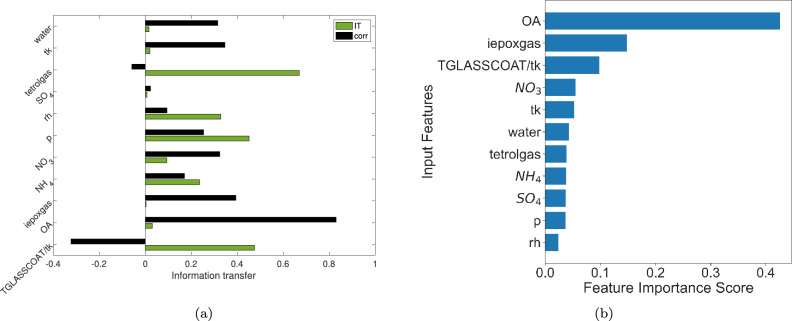
(**a**) Information transfer to total tetrol and correlation of total tetrol with other variables for altitudes of 10–12 km. (**b**) Feature importance identified by Random Forest for total tetrol for altitudes of 10–12 km.

We compute the information transfer between the various chemical species at these two levels and the details of the information transfer computation algorithm are described in Section 3B of the Supplementary Material. We also compare and contrast the information transfer measure with correlation and the Random Forest (RF) techniques and the results are presented in Figs. 5 and 6.

### Key influential variables affecting tetrol particles

Figure 5a shows the influence and correlation of the various variables with total tetrol particles (sum of semi-volatile tetrol in particle phase and tetrol oligomer). Note that tetrol oligomers are formed from particle-phase oligomerization of tetrol particles; hence, we add tetrol particles and tetrol oligomers. It can be seen that $SO_4$ has the highest information transfer to total tetrol, followed by water and $NO_3$. In comparison, Fig. 5b shows the feature importance of different independent variables in predicting total tetrol particle concentrations, as identified by the Random Forest (RF) algorithm. Although information transfer identifies $SO_4$ and water as the most influential variables, RF identifies *OA* as the most important feature, followed by iepoxgas (Fig. 5b). $SO_4$ and water are key nucleophiles that affect the ring opening of IEPOX and particle-phase acidity, which are essential for the formation of particle-phase IEPOX-SOA. The information transfer predictions are consistent with our domain knowledge of the multiphase chemistry of IEPOX-SOA. In contrast, the RF identified features *OA* and IEPOX gas as the most important features and this is similar to the correlation of the variables with IEPOX-SOA, as shown by the red bars in Fig. 5a. IEPOX gas is necessary to produce IEPOX-SOA; therefore, the RF identification of IEPOX gas as a key feature contributing to IEPOX-SOA is consistent with our understanding, which is also reflected in its highest correlation with total tetrol particles. However, the information transfer from IEPOX gas to total tetrol is low (green bar in Fig. 5a). This is likely because IEPOX gas does not directly transfer information to IEPOX-SOA, and the information is transferred through other variables like water and RH (discussed later). This effect can be understood by the example system (1). Here $x_2$ and $x_5$ are correlated and RF identifies $x_2$ having non-zero feature importance as far as prediction of $x_5$ is concerned. However, we see that there is no direct causal link from $x_2$ to $x_5$ (see Fig. 3a), but $x_2$ *influences* $x_5$ via $x_4$. In other words, $x_2$ has an indirect influence on $x_5$. Similarly, IEPOX gas has an indirect influence on IEPOX-SOA. Note that although acidity is known to influence IEPOX-SOA formation, we did not include it as a separate feature since acidity depends on other features like $SO_4$ and particle water. In addition, particle acidity, water and sulfate have been reported to have convoluted interactions, especially in regions like the southeast USA where particle acidity and water contents are already high and are not limiting IEPOX-SOA formation (similar to the Amazon where it is humid and particles are acidic)^22^. In field experiments, particle sulfate was reported to have most direct causal relations with IEPOX-SOA formation^22^. Moreover, in field experiments acidity is not measured but diagnosed from thermodynamic models as a function of particle water and its inorganic content, therefore, the particle inorganic content and water included in our analysis implicitly include the effects of acidity. The information transfer approach correctly identifies particle sulfate and water as the variables that directly affect IEPOX-SOA formation, consistent with their known effects on particle phase chemistry. Again, since IEPOX-SOA is a part of the total OA, it is no surprise that these are correlated.

We also analyzed the causal structure for tetrol particles at higher altitudes, i.e., at altitudes of 10-12 km in the upper troposphere over the Amazon. While several features influence tetrol particles near the surface due to atmospheric multiphase chemistry (chemistry in gas-phase and liquid water associated with aerosols and clouds), aqueous chemistry is shut off in the upper troposphere due to extremely cold temperatures and a lack of liquid water in aerosols and clouds at high altitudes^6^. Tetrol particles at high altitudes are mostly transported by clouds in the gas phase, followed by the condensation of the semi-volatile tetrol gases at cold temperatures (225 K) in the upper troposphere^6^. Our information transfer analysis identified this process included in WRF-Chem related to the condensation of tetrol gases as a key source of IEPOX-SOA in the upper troposphere. Fig. 6a shows that at higher altitudes, tetrolgas has the highest influence on total tetrol, followed by TGLASSCOAT/tk, which is a direct predictor of bulk viscosity, and diffusion limitations that limit the formation of IEPOX-SOA. Therefore, while tetrol gas is a direct source of tetrol particles, increases in TGLASSCOAT/tk (and corresponding increases in OA viscosity) are expected to reduce tetrol particle formation by decreasing the formation of IEPOX-SOA (shown by the inverse correlation between tetrol particles and TGLASSCOAT/tk in Fig. 6a).

In contrast to the information transfer analyses described above, a random forest (RF) analysis identifies OA as the most important feature (Fig. 6b) influencing tetrol particles at high altitudes (similar to the near surface case shown in Fig. 5), which can be explained by their correlations (Fig. 6a). This indicates that the feature importance determined by RF mostly identifies correlated variables but is not a necessary indicator of causality. Since precursors of IEPOX-SOA like tetrol gases and OA components are all transported by clouds to the upper troposphere, it is reasonable to expect correlation between OA and IEPOX-SOA. However, it is encouraging that the causal approach could identify tetrol gases as causally related to IEPOX-SOA. As shown in our previous study^6^, turning off convective transport of tetrol gases and other chemical components from the surface to the upper troposphere within WRF-Chem resulted in an order of magnitude decrease in simulated IEPOX-SOA in the upper troposphere, which clearly established that the major source of total tetrol particles (dominant IEPOX-SOA components) in the upper troposphere was transport of tetrol gases by clouds followed by their condensation at cold temperatures to form IEPOX-SOA particles. This causal link between tetrol gases and total tetrol particles in the upper troposphere is uniquely indentified just by the causal approach.

### Influence graph and causation v/s correlation

In addition to causality, information transfer analyses could also be used to qualitatively analyze how certain variables are correlated through an influence graph of the underlying system.

#### Definition 1

(*Influence Graph*) An influence graph for a dynamical system $x(t+1)=T(x(t))$, with states $x(t) = [x_1(t), x_2(t), \cdots , x_n(t)]^\top \in {\mathbb {R}}^n$, is a weighted directed graph $\mathcal{G}(t)=\{(V(t),E(t),W(t))| V(t) = \{1,2,\cdots , n\}; E(t) = (i,j)\subseteq V\times V; W(t) (= [w_{ij}(t)]_{i,j=1}^n) \in {\mathbb {R}}^{n\times n} \}$, where the node set *V*(*t*) correspond to the dynamic states, *E*(*t*) is the set of directed edges such that there is a directed edge $(i,j)\in E(t)$ from node *i* to node *j* if and only if the information transfer from $x_i(t)$ to $x_j(t)$ is non-zero at time *t* and the $ij^{th}$ entry $w_{ij}(t)$ of the weight matrix *W*(*t*) is equal to the absolute value of the information transfer from state $x_i(t)$ to state $x_j(t)$ at time *t*.

Note that, in general, an influence graph can have both a time-varying topology and time-varying weights. The topology of the influence graph will change if the underlying system topology is time-varying. On the other hand, the information transfer values (and hence the edge-weights of the influence graph) can change with time during transient dynamics, also resulting in a time-varying influence graph. But as mentioned earlier, the steady-state information transfer values for stable systems attain a steady state, resulting in a unique influence graph. In the context of this work, while different features affecting tetrol particles are time- and space-varying, they originate from stable processes, and we consider the steady-state information transfer values in constructing the influence graphs.

By definition, the directed edges of an influence graph give the direct causal links present in the system. However, it can be used to identify indirect causal links and also the confounding variables. For example, the influence graph of system (1) is exactly the same as Fig. 3a and hence from the influence graph one can identify the confounding variable, which explains the high correlation of the states $x_3$ and $x_5$, even though they are not causally connected. Furthermore, as mentioned earlier, RF identifies important features, but it captures a sort of statistical dependence and that is why it identified $x_3$ and $x_4$ as the most important features for predicting $x_5$. However, there is no causal connection between $x_3$ and $x_5$ (see Fig. 3c), which our influence graph clearly shows and thus the importance of the influence graph lies in the fact that it identifies indirect influences and confounding variables.

**Figure 7:**
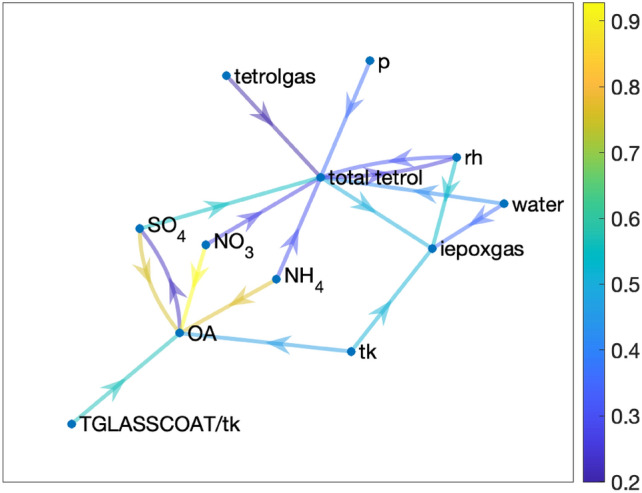
Causal structure for total tetrol for altitude 0–500 m.

Fig. 7 shows the influence graph for total tetrol particles near the surface. For the sake of clarity, we only show the most influential variables and ignore the variables with low information transfers to tetrol particles. The importance of the influence graph lies in the fact that it can be used to infer indirect influence and statistical dependence. For example, Fig. 5a shows that the information transfers from both iepoxgas and OA to total tetrol are low, whereas the correlation of these variables with total tetrol is high, likely because tetrol particles are a part of OA (that explains why they are correlated). In addition, IEPOX gas is a necessary precursor to tetrol particle formation but is not sufficient by itself since the formation of tetrol particles (a key IEPOX-SOA component) depends on other factors as well, including particle water and acidity. Both particle water content and acidity strongly depend on inorganic components of particles, including rh, particle $NO_3$, $NH_4$ and $SO_4$ that govern particle hygroscopicity and water uptake (in addition to OA). Consistent with this known chemistry, the influence graph of Fig. 7 shows that OA is related to total tetrol mostly via $NO_3$, $NH_4$ and *tk*. *tk* affects the gas-to-particle partitioning of tetrols. Hence, even though the information transfer from OA to total tetrol is small, they form connected components of the influence graph and thus this leads to a correlation between OA and total tetrol. (This difference in correlation and information transfer approaches between OA and total terol particles is analogous to our simple example showing how states $x_2$ and $x_5$ are correlated in system (1) but information flow from $x_2$ to $x_5$ is zero. The observed correlation between $x_2$ and $x_5$ is mainly because of $x_2$ influencing $x_5$ via $x_4$ (see Fig. 3a)). Similarly, iepoxgas is related to total tetrol mostly through OA, water and rh, and hence they are correlated. This is a key validation of the information transfer approach and its ability to identify many of these known complex causal relations between key features affecting tetrol particles, as described above.

**Figure 8:**
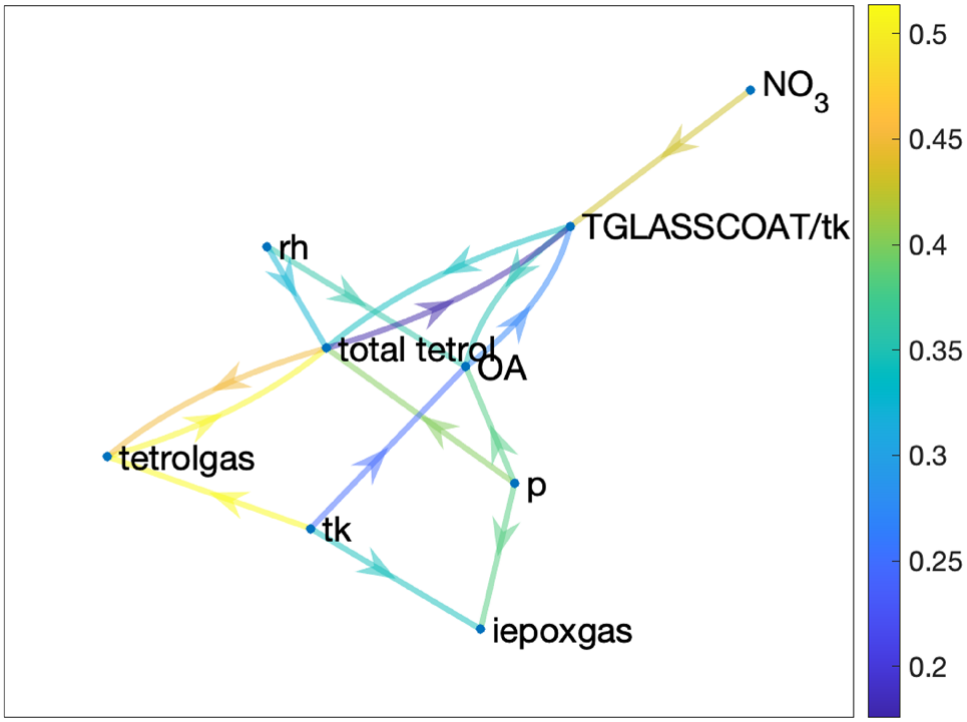
Causal structure for total tetrol for altitude 10–12 km.

Figure 8 presents the influence graph for tetrol particles at altitudes of 10–12 km. Compared to the densely connected near surface case (Fig. 7), we find that the influence graph for total tetrol in the upper troposphere is sparsely connected. For example, $SO_4$ is not a key influential variable at higher altitudes, whereas near the surface, $SO_4$ was identified as a key variable influencing tetrol particles. Similarly, information transfer determines that $NH_4$ and $NO_3$ are not significant variables influencing tetrols in the upper troposphere. This is consistent with our domain knowledge that due to low RH and cold temperatures in the upper troposphere, both inorganic ($SO_4$, $NO_3$ and $NH_4$) and organic (OA) components of particles are in the solid phase and thereby lack particle water, shutting off the aqueous chemistry needed for in-situ IEPOX-SOA and tetrol particle formation at high altitudes. Moreover, we also find that at higher altitudes, OA has the highest correlation with total tetrol, whereas at lower altitudes, iepoxgas has the highest correlation with total tetrol. However, similar to the low altitude case, one can explain the correlation of tetrol particles with OA from Fig. 8, where we can see that tetrol particles and OA are related through tk, TGLASSCOAT/tk and tetrolgas, which explains the high correlation between tetrol particles and OA. But the information transfer analysis correctly identifies that the key causal feature affecting tetrol particles at high altitudes is tetrol gas (tetrol in the gas phase) that condenses at low temperatures (low tk) to form tetrol particles (tetrol in the particle phase). In addition, TGLASSCOAT/tk is also identified as a causal feature since an increase in TGLASSCOAT/tk reduces the aqueous chemistry source of tetrol particles further as they are transported to higher altitudes.

While, in this study, we had pre-existing domain knowledge of features affecting IEPOX-SOA to evaluate RF and information transfer analyses, there are many instances where features affecting a given variable of interest might not be known a priori, e.g., in field measurements. In such cases, correlation and RF analyses might be useful as first steps to determining the key features of interest. However, subsequently, information transfer analysis, as depicted in this work, will be instrumental in further analyses for the determination of causality and the differentiation between correlated and causal features.

### Causal structure in presence of noise

One of the main concerns for any data-driven technique is how robust the framework is to the presence of noise in the data, because real world data is almost always corrupted with noise. The information transfer theory is applicable to stochastic dynamical systems with additive noise^3,4^ and while computing the information transfer measure from data, we use the Robust Koopman Operator theory^23,24^ which inherently takes into account both process and/or measurement noise. Thus, the information transfer measure is robust to noise in the data. However, realistically, if the noise is comparable to the amplitudes of the state values (i.e. if signal and noise are comparable in magnitudes), then neither Robust Koopman or the information transfer conclusions can be trusted. But this is true for any learning algorithm.

The data used in this paper was obtained from the WRF-Chem model and since it is simulated data it is not affected by measurement noise. Although we used modeling data in this work, our modeled WRF-Chem outputs have been extensively validated with field measurements as documented in our previous study^6^. In this sense, it is expected that our modeled outputs represent continuous and smooth representations of actual ambient measurements. However, to examine the influence of noise, we added independent and identically distributed (i.i.d.) Gaussian noise with zero mean and variance of 0.2 and looked at the influence of the different features on total tetrol particle (Note: total terol particles are dominant components of IEPOX-SOA) concentrations near the surface i.e. at 0-500m altitudes at each time point. The results are shown in Supplementary Fig. S6. We find that for noise-free data, the information transfer algorithm identifies $SO_4$ and particle water as the most influential variables affecting tetrol particles. With added noise $SO_4$ and iepoxgas are the two variables with highest IT values, followed by particle water. Near the surface, IEPOX gas is the key precursor that needs to be reactively taken up by particles to form IEPOX-SOA, but it is not sufficient by itself to form IEPOX-SOA since particle phase sulfate and water are also needed. Therefore, the identification of IEPOX gas when noise is introduced is still consistent with our domain knowledge of IEPOX-SOA (comprising mostly total tetrol particles). Addition of noise to all variables somewhat reduces the signal to noise ratio and the results of IT. However, in the absence of noise (Fig. S6a), the information transfer from IEPOX gas to total tetrols is low. This is likely because the IT approach identifies iepoxgas is related to total tetrol mostly through OA, water and rh as shown in Fig. 7. However, the relative IT from other less influential variables to tetrol particles changes when noise is added to the same dataset.

### Causal structure in presence of additional extraneous variables

In many real world examples, often, it is not possible to obtain the measurements of all the states. Additionally, for complicated systems one may not even know how many dynamic states there are in the system. In this paper we considered only a subset of the dynamic variables of the WRF-Chem Model that are known to directly influence the chemistry of IEPOX-SOA formation. However, if we had considered a larger set of extraneous variables (related to the original 11 input variables but not completely independent of those variables), the causal structure may change. We performed additional causal analyses with 18 input variables (chemical and transport related variables) instead of 11 variables and the results are presented in the Supplementary Material (See Supplementary Fig. S7). It can be seen that for both 18 and 11 input variable scenarios, our information transfer algorithm identifies $SO_4$ as the most influential variable affecting total tetrol particles. However, when 18 variables are considered, the horizontal and vertical velocities (U, V, W) also show up as influential variables, which is consistent with the expectation that total tetrol particles are affected by transport and mixing, in addition to chemistry.

In summary, it is encouraging that adding extraneous variables related to chemistry ($O_3$, *NO*, $NO_2$, *OH* radicals) and horizontal velocity (U,V) and vertical velocity W to the existing set of 11 input variables does not change the dominant feature ($SO_4$) affecting total tetrol particles near the surface. However, the IT identifies additional variables i.e. horizontal and vertical velocity components affecting transport and mixing of IEPOX-SOA near the surface.

## Discussions

A lot of useful insights could be gained by examining the dynamic time series of meteorological and chemical variables in field measurements that might affect atmospheric chemistry and fine particle formation. However, correlations inferred from these measurements cannot be directly used to infer causal relations between key variables of interest because of the role of confounding variables and indirect relations between features. Here, we use one of the most advanced data-driven approaches: the Koopman operator framework and information transfer analyses to investigate causal relationships within the atmospheric chemistry pathways that lead to fine particle formation. We focus on 2-methyltetrols, which are often the major constituents of IEPOX-SOA that is formed by multiphase chemistry.

The data from the WRF-Chem modeled IEPOX-SOA and related features that we use in this study have been evaluated with a suite of field-based aircraft measurement data over the Amazon in our previous study^6^. Based on the model-measurement comparison, new processes related to surface and in plant biochemistry that emit 2-methyltetrol gases were identified and incorporated into WRF-Chem. Therefore, the modeled data, which we use in this study, implicitly represents observed atmospheric chemistry, and further it represents spatially and temporally continuous variable dynamics needed for our study. In addition, to the best of our knowledge, this study represents a first proof of concept application of Koopman framework to atmospheric chemistry and aqueous SOA formation. It was necessary to select a system where the causal processes are known for evaluating our approach, and we believe our WRF-Chem model that has already been evaluated with measurements was an ideal choice.

We find that near the Earth’s surface, the information transfer approach determined particle water and sulfate as the dominant features affecting particle-phase tetrols, consistent with the domain knowledge of the role of these variables in the formation of IEPOX-SOA. In contrast, RF and correlation analyses identified OA as the key feature governing IEPOX-SOA, but causal analyses showed a much smaller direct information transfer from OA to tetrols. Further analyses using influence graphs with information transfer showed that OA is indirectly related to tetrol particles via other variables, including $NO_3$, $NH_4$ and *tk*, which causes a high correlation between OA and tetrols, but this correlation does not imply direct causality. In contrast, at high altitudes (in the upper troposphere), the information transfer analyses showed that semi-volatile tetrol gases were most important for tetrol particles. Again, this causal relation between tetrol gases and particles at high altitudes is consistent with the process included in WRF-Chem about the transport of tetrol gases to high altitudes by clouds and their condensation to form tetrol particles at high altitudes, where aqueous atmospheric chemistry is much less important compared to near-surface^6^. Note that our causal analysis approach deduced these key contrasting processes between the surface and upper troposphere just by analyzing the time series data of the variables listed in Table 1, while RF analysis of the same dataset identified correlated features that were similar between the surface and upper troposphere.

We show that results from the three methods (RF, correlation, IT) are very different. This is an important result since most studies might just use one of these methods, and it is not known which method is right. As explained above, we use our domain knowledge and WRF-Chem simulated IEPOX-SOA processes that are different near the surface and upper troposphere to provide insights about the utility of our three different approaches for causal analysis. We find that the IT process is successful in atleast identifying the dominat feature affecting IEPOX-SOA under different regimes: near surface versus upper troposphere. However, for the other less important features, it is difficult to accurately assign their IT to IEPOX-SOA. This is mainly because the IEPOX-SOA system is governed by coupled non-linear processes, and several variables/features are not completely independent. In addition, we show that even with inclusion of noise and additional extraneous variables, the IT approach identifies the dominant features affecting IEPOX-SOA as long as the signal-to-noise ratio is high. With additional extraneous variables related to chemistry and meteorology that are not completely independent of the original 11 variables, the IT approach identified horizontal and vertical wind components as additional influential variables affecting IEPOX-SOA near the surface. Thus, we show that the IT approach provides useful information about interconnections between these complex features.

Our study provides the first evidence that the Koopman framework with an information transfer approach could be used to gain insights into causal relations between key variables of interest just by analyzing their time series from detailed regional model analyses. Our work has tremendous implications for the analyses of measurements and models, could be used to understand unknown processes that might affect variables of interest, and could likely be applied in diverse domains, e.g., climate, air quality and human health, to identify unknown causal relations.

## Methods

### WRF-Chem model

The Weather Research and Forecasting Model Coupled to Chemistry (WRF-Chem) is a community three-dimensional chemical transport model that couples clouds, gas-phase and particle-phase chemistry, meteorology and radiation online and interactively^25^. The model is used to study coupled physical and chemical processes such as aerosol-cloud interactions, clouds and convection.

The WRF-Chem model was run at a moderately high resolution of 10 km grid spacing encompassing a $1500\times 1000$ km domain from near-surface to the free troposphere (altitudes of 15 km) for September 22–30, 2014, over the Amazon rain forest to simulate the formation of secondary organic aerosol (SOA)^6^ and the dynamic variables considered in this paper are tabulated in Table 1. The vertical altitude range of 0–15 km is represented by 45 vertical levels, with half the number of vertical levels placed at the lowest 2 km altitude. SOA refers to a class of thousands of organic compounds formed in the atmosphere by the oxidation of organic gases emitted by plants and combustion activities include anthropogenic and biomass burning emissions. In the Amazon rain forest, SOA is the dominant contributor to a fine particular matter and its formation is governed by complex interactions between chemistry, clouds and meteorology. We simulate the multiphase chemical pathways in gas-phase, particle-phase and clouds governing SOA formation in WRF-Chem using a combination of approaches. Interested readers can find further details about the modeling in^6^. WRF-Chem outputs are spatio-temporal data for hundreds of variables, including different meteorological and chemical features. Here, we focus on assessing the causal relationships between the formation of particle-phase IEPOX-SOA, which includes semi-volatile 2-methyltetrols (tetrol) that can exist in both gas- and particle-phase, and non-volatile tetrol oligomers (tetolig). Mathematical equations in the aqueous chemistry module within WRF-Chem define the relationships between these variables of interest and other variables, as documented in^6^. Here, our objective is to determine whether this causality can be inferred from the time series outputs of key variables.

### Information transfer computation

We used a total of 216 output files (hourly data) for each grid cell from WRF-Chem predictions for our causal analyses. For the computation of the finite-dimensional approximation of the Robust Koopman Operator^23^, we chose linear dictionary functions with the regularization parameter $\lambda$ set to 0.01 for all the simulations. The choice of $\lambda$ is governed by the fact that the Koopman operator is a Markov operator and has at least one unit eigenvalue^26^. In particular, for Hamiltonian systems, all the eigenvalues are equal to one, but for dissipative systems, it always has at least one unit eigenvalue and we choose that particular $\lambda$ for which the computed Koopman operator has at least one unit eigenvalue. We note that for both the lower and upper altitudes, the $\lambda$ remains the same, that is, $\lambda = 0.01$. Once the original and frozen dynamics were learned by the Koopman operator, we used the closed form expressions for information transfer to obtain the information transfer values between the different variables. The details of data-driven information transfer computation are described in the Supplementary Material.

### Supplementary Information

Supplementary Information.

## Data Availability

All data analyzed during the current study are included in this published article and its Supplementary Material. The causal inference code and outputs from WRF-Chem that are used to generate figures in this study are available from the corresponding author on reasonable request.

## Code availability

All codes used for analyses are available upon reasonable request.

## References

[1] Seinfeld JH, Bretherton C, Carslaw KS, Coe H, DeMott PJ, Dunlea EJ, Feingold G, Ghan S, Guenther AB, Kahn R (2016). Improving our fundamental understanding of the role of aerosol- cloud interactions in the climate system. Proc. Natl. Acad. Sci..

[2] Shrivastava M, Cappa CD, Fan J, Goldstein AH, Guenther AB, Jimenez JL, Kuang C, Laskin A, Martin ST, Ng NL (2017). Recent advances in understanding secondary organic aerosol: Implications for global climate forcing. Rev. Geophys..

[3] Sinha S, Vaidya U, Sinha S, Vaidya U (2016). Causality preserving information transfer measure for control dynamical system. 2016 IEEE 55th Conference on Decision and Control (CDC).

[4] Sinha S, Vaidya U, Sinha S, Vaidya U (2017). On information transfer in discrete dynamical systems. 2017 Indian Control Conference (ICC).

[5] Sinha S, Vaidya U (2020). On data-driven computation of information transfer for causal inference in discrete-time dynamical systems. J. Nonlinear Sci..

[6] Shrivastava M, Rasool QZ, Zhao B, Octaviani M, Zaveri RA, Zelenyuk A, Gaudet B, Liu Y, Shilling JE, Schneider J (2022). Tight coupling of surface and in-plant biochemistry and convection governs key fine particulate components over the amazon rainforest. ACS Earth Space Chem..

[7] Granger CWJ (1969). Investigating causal relations by econometrics models and cross-spectral methods. Econometrica.

[8] Granger CWJ (1980). Testing for causality. J. Econ. Dyn. Control.

[9] Shannon CE (1948). A mathematical theory of communication. Bell Syst. Tech. J..

[10] Massey, J. L. Causality, feedback and directed information, in: Proc. Intl. Symp. on Info. th. and its Applications, Waikiki, Hawai, USA, pp. 303–305 (1990).

[11] Schreiber T (2000). Measuring information transfer. Phys. Rev. Lett..

[12] Breiman L (2001). Random forests. Mach. Learn..

[13] Sinha S, Sharma P, Vaidya U, Ajjarapu V, Sinha S (2017). Identifying causal interaction in power system: Information-based approach. 2017 IEEE 56th Annual Conference on Decision and Control (CDC).

[14] Sinha S, Sharma P, Vaidya U, Ajjarapu V (2019). On information transfer-based characterization of power system stability. IEEE Trans. Power Syst..

[15] Sinha S, Vaidya U (2018). Data-driven approach for inferencing causality and network topology. Annu. Am. Control Conf. (ACC).

[16] Marko H (1973). The bidirectional communication theory—A generalization of information theory. IEEE Trans. Commun..

[17] Lasota A, Mackey MC (1994). Chaos, Fractals, and Noise: Stochastic Aspects of Dynamics.

[18] Budisic M, Mohr R, Mezic I (2012). Applied koopmanism. Chaos.

[19] Sinha S, Nandanoori SP, Yeung E (2020). Koopman operator methods for global phase space exploration of equivariant dynamical systems. IFAC-PapersOnLine.

[20] Williams MO, Kevrekidis IG, Rowley CW (2015). A data-driven approximation of the koopman operator: Extending dynamic mode decomposition. J. Nonlinear Sci..

[21] Louppe, G., Wehenkel, L., Sutera, A. & Geurts, P. Understanding variable importances in forests of randomized trees. Adv. Neural Inf. Process. Syst. 26 (2013).

[22] Xu L, Guo H, Boyd CM, Klein M, Bougiatioti A, Cerully KM, Hite JR, Isaacman-VanWertz G, Kreisberg NM, Knote C (2015). Effects of anthropogenic emissions on aerosol formation from isoprene and monoterpenes in the southeastern united states. Proc. Natl. Acad. Sci..

[23] Sinha S, Huang B, Vaidya U (2020). On robust computation of koopman operator and prediction in random dynamical systems. J. Nonlinear Sci..

[24] Sinha S, Nandanoori SP, Barajas-Solano D (2023). Online real-time learning of dynamical systems from noisy streaming data. Nat. Sci. Rep..

[25] Grell GA, Peckham SE, Schmitz R, McKeen SA, Frost G, Skamarock WC, Eder B (2005). Fully coupled “online” chemistry within the wrf model. Atmos. Environ..

[26] Budišić M, Mohr R, Mezić I (2012). Applied Koopmanism. Chaos Interdiscip. J. Nonlinear Sci..

