# Supplementary material for: Application of advanced causal analyses to identify processes governing secondary organic aerosols
Source: Sci Rep. 2024 May 10;14:10718. doi: 10.1038/s41598-024-59887-7 (PMC11087645; doi:10.1038/s41598-024-59887-7)
Supplement: Supplementary file 1 — Supplementary Information. [file 41598_2024_59887_MOESM1_ESM.pdf]

Supplemental Material

Information Transfer in Dynamical Systems

In this section, we briefly describe the concept of information transfer in a dynamical system. For details, see [1, 2].

Probability, Disorder and Entropy

In this work, by information, we mean the Shannon information of a probability distribution [3]. In particular, given a continuous probability distribution $\rho(x)$, defined on an event space Ω, the Shannon entropy of the distribution is given by

$$H(x) = -\int_{\Omega} \rho(x) \log \rho(x) dx. \tag{1}$$

For a discrete distribution $p_i(x)$, $i = 1, 2, \cdots, n$, the entropy is given by

$$H(x) = -\sum_{i=1}^{n} p_i(x) \log p_i(x). \tag{2}$$

For example, consider the event of coin flips where the coin is biased and let $X = 1$ represent the result of heads. Fig. S1 shows the entropy of the process as a function of the probability of getting heads. When the coin is heavily biased, for example, towards getting a head, then even before the coin is flipped, one can say with a high degree of certainty that the outcome of the flip will be a head. Hence, there is very *low uncertainty* in the outcome of the experiment, and hence the Shannon entropy is low in this case. On the other hand, when the coin is completely unbiased, there is a 50% chance of getting a head and a 50% chance of getting a tail. So there is *maximum uncertainty* in the outcome, and hence, at this probability, $Pr(X = \frac{1}{2})$, the entropy is a maximum. So, in this sense, Shannon entropy is a measure of uncertainty.

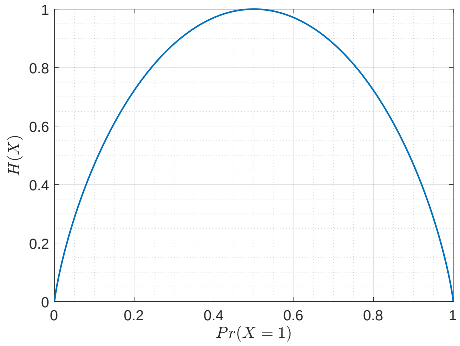

Figure S1: Entropy $H(X)$ of a coin flip experiment of a biased coin as a function of the bias $Pr(X = 1)$. Here, $X = 1$ represents the result of heads.

On the other hand, Ludwig Boltzmann described the entropy of a statistical mechanical system as the number of microstates that lead to the same macrostate [4, 5]. For example, consider a system of gas in a box. Then the volume, pressure and temperature describe the macrostates of the system. However,

microscopically, the system consists of a large number of molecules all moving around and colliding with each other and with the walls of the cylinder, with the microstates being the position and momenta of the molecules. Boltzmann entropy connects the world of the microstates with that of the macrostates, and this relation is regarded as the birth of the field of statistical mechanics. In particular, given a macrostate, Boltzmann entropy counts the number of distinct microstates that lead to the given macrostate. Hence, the larger the number of microstates leading to the macrostate, the larger the entropy. In other words, if there are a large number of admissible positions and momenta that lead to a given macrostate, it means that the molecules of the gas have a large number of choices of position and momenta to choose their own microstates from, and thus the microstates can be more disorderly. Hence, in this sense, Boltzmann entropy is a measure of the disorder of a system. And since higher disorder usually means higher uncertainty, Boltzmann entropy and Shannon entropy qualitatively describe similar things about a system.

Information Transfer in a Dynamical System

In this subsection, we briefly describe the concept of information transfer in a dynamical system. For details, we refer the reader to [1, 2]. Consider a dynamical system,

$$z(t+1) = f(z(t)) + \xi(t), \tag{3}$$

where $z = [x^\top \quad y^\top]^\top \in \mathbb{R}^n$, $f = [f_x^\top \quad f_y^\top]^\top$, $x \in \mathbb{R}^{|x|}$, $y \in \mathbb{R}^{|y|}$ (here $|\cdot|$ denote the dimension of $\{\cdot\}$), $f_x : \mathbb{R}^{|x|+|y|} \to \mathbb{R}^{|x|}$ and $f_y : \mathbb{R}^{|x|+|y|} \to \mathbb{R}^{|y|}$ are assumed to be at least continuously differentiable and $\xi(t) = [\xi_x(t)^\top, \xi_y(t)^\top]^\top$ is independent and identically distributed additive noise. Furthermore, let $\rho(z(t))$ denote the distribution of the states z at time t and let $\rho(z(t+1)|z(t))$ denote the conditional distribution of $z(t+1)$ conditioned on $z(t)$.

Next, given the system of the form (3), consider a modified dynamical system where the x dynamics have been held constant from time step t to time step $t+1$, that is, the dynamical system

$$\left.\begin{array}{rcl} x(t+1) & = & x(t) \\ y(t+1) & = & F_y(x(t), y(t)) \ + \xi_y(t) \end{array}\right\} = f_{\not x}(z(t)) + \xi_{\not x}(t). \tag{4}$$

For this modified system, we denote by $\rho_{\not x}(y(t+1)|y(t))$ the probability density function of $y(t+1)$ conditional on $y(t)$, with the dynamics in x coordinate frozen in time from time step t to $t+1$ as in Eq. (4). With this, we have the following definition of information transfer from $x \to y$, as the system (3) evolves from time step t to $t+1$.

Definition 1. *[Information transfer] [1, 2] The information transfer from x to y for the dynamical system (3), as the system evolves from time t to time $t+1$ (denoted by $[T_{x\to y}]_t^{t+1}$), is given by the following formula:*

$$[T_{x\to y}]_t^{t+1} = H(\rho(y(t+1)|y(t))) - H(\rho_{\not x}(y(t+1)|y(t)), \tag{5}$$

where $H(\rho(y)) = -\int_{\mathbb{R}^{|y|}} \rho(y)\log\rho(y)dy$ is the entropy of the probability density function $\rho(y)$ and $H(\rho_{\not x}(y(t+1)|y(t))$ is the entropy of $y(t+1)$, conditioned on $y(t)$, where x has been frozen as in Eq. (4).

The intuition behind the definition of information transfer is the fact that the total entropy of the variable y can be decomposed as the sum of the entropy of y when x is not present in the system (held constant as in Eq. (4)) and the entropy transferred from x to y. The information transfer from x to y depicts how the evolution of x affects the evolution of y, that is, it gives a quantitative measurement of the influence of x on y and is thus inherently asymmetric. With this, we have the following definition of influence:

Definition 2. *[Influence] A state (or subspace) x influences a state (or subspace) y if and only if the information transfer from x to y is non-zero. Furthermore, the quantitative measure of the influence $[I_{x\to y}]_t^{t+1}$*

of the state (subspace) x on state (subspace) y, as the system (3) evolves from time step t to time step $t+1$, is given by the absolute value of the information transfer from x to y at time step t, that is

$$[I_{x\to y}]_t^{t+1} = |[T_{x\to y}]_t^{t+1}| = |H(\rho(y(t+1)|y(t))) - H(\rho_{\not{x}}(y(t+1)|y(t))|. \tag{6}$$

Information Transfer in Linear Dynamical Systems

The computation of the information transfer measure requires us to track the evolution of the probability density of the states, and this evolution is given by the Perron-Frobenius (P-F) operator [6]. However, for general nonlinear systems, there is no closed-form expression for the evolution of entropy, and as such, one has to resort to numerical techniques for computing the information transfer. However, for linear systems with i.i.d. Gaussian noise, one can derive a closed-form expression for information transfer. For a linear dynamical system of the form,

$$z(t+1) = Az(t) + \sigma\xi(t) \tag{7}$$

where $z(t) \in \mathbb{R}^N$, $\xi(t)$ is vector-valued Gaussian random variable with zero mean and unit variance, and $\sigma > 0$ is a constant, the information transfer from any state (subspace) x_1 to y is given by [1, 2]

$$[T_{x_1\to y}]_t^{t+1} = \frac{1}{2}\log\frac{|A_{yx}\Sigma_y^s(t)A_{yx}^\top + \sigma^2 I|}{|A_{yx_2}(\Sigma_y^s)_{yx_2}(t)A_{yx_2}^\top + \sigma^2 I|}, \tag{8}$$

where $z^\top = (x^\top, y^\top)^\top = (x_1^\top, x_2^\top, y^\top)^\top$,

$$A = \begin{pmatrix} A_x & A_{xy} \\ A_{yx} & A_y \end{pmatrix} = \begin{pmatrix} A_{x_1} & A_{x_1x_2} & A_{x_1y} \\ A_{x_2x_1} & A_{x_2} & A_{x_2y} \\ A_{yx_1} & A_{yx_2} & A_y \end{pmatrix}, \tag{9}$$

$$\Sigma = \begin{pmatrix} \Sigma_x & \Sigma_{xy} \\ \Sigma_{xy}^\top & \Sigma_y \end{pmatrix} = \begin{pmatrix} \Sigma_{x_1} & \Sigma_{x_1x_2} & \Sigma_{x_1y} \\ \Sigma_{x_1x_2}^\top & \Sigma_{x_2} & \Sigma_{x_2y} \\ \Sigma_{x_1y}^\top & \Sigma_{x_2y}^\top & \Sigma_y \end{pmatrix}, \tag{10}$$

$$\Sigma(t) = A\Sigma(t-1)A^\top + \sigma^2 I, \tag{11}$$

$\Sigma_y^s(t) = \Sigma_x(t) - \Sigma_{xy}(t)\Sigma_y(t)^{-1}\Sigma_{xy}(t)^\top$ is the Schur complement of $\Sigma_y(t)$ in the matrix $\Sigma(t)$ and $(\Sigma_y^s)_{yx_2}$ is the Schur complement of Σ_y in the matrix

$$\Sigma_y = \begin{pmatrix} \Sigma_{x_2} & \Sigma_{x_2y} \\ \Sigma_{x_2y}^\top & \Sigma_y \end{pmatrix}.$$

The general expression for information transfer between scalar states z_i and z_j can be obtained by substituting z_i, z_j and their complements accordingly in (8).

For linear systems with Gaussian noise, the one-step zero transfer can be characterized by looking at the system matrix A. In particular, we have the following theorem:

Theorem 3. *For a linear system with system matrix A, $A_{z_jz_i} = 0$ if and only if $[T_{z_i\to z_j}]_t^{t+1} = 0$ for all $t \in \mathbb{Z}_{\geq 0}$.*

For details, see [1, 2].

Transfer Operators for Dynamical Systems

In this section, we briefly describe the basics of transfer operator theory for a discrete-time dynamical system. For more details, see [6].

Consider a discrete-time dynamical system

$$x_{t+1} = T(x_t) \tag{12}$$

where $T : X \subset \mathbb{R}^N \to Z$ is assumed to be at least of class $\mathcal{C}^1$. Associated with the dynamical system (12) is the Borel-σ algebra $\mathcal{B}(X)$ on X and the vector space $\mathcal{M}(X)$ of bounded complex-valued measures on X. With this, two linear operators, namely, the Perron-Frobenius (P-F) and the Koopman operator, can be defined as follows [6]:

Definition 4 (Perron-Frobenius Operator). *The Perron-Frobenius operator* $\mathbb{P} : \mathcal{M}(X) \to \mathcal{M}(X)$ *is given by*

$$[\mathbb{P}\mu](A) = \int_X \delta_{T(x)}(A) d\mu(x) = \mu(T^{-1}(A)),$$

where $\delta_{T(x)}(A)$ *is the stochastic transition function that measures the probability that point* x *will reach the set* A *in one-time step under the system mapping* T*.*

Definition 5 (Invariant measures). *Invariant measures are the fixed points of the P-F operator* $\mathbb{P}$ *that are also probability measures. Let* $\bar{\mu}$ *be the invariant measure then,* $\bar{\mu}$ *satisfies*

$$\mathbb{P}\bar{\mu} = \bar{\mu}.$$

If the state space Z is compact, it is known that the P-F operator admits at least one invariant measure.

Definition 6 (Koopman Operator). *Given any* $h \in \mathcal{F}$, $\mathbb{U} : \mathcal{F} \to \mathcal{F}$ *is defined by*

$$[\mathbb{U}h](x) = h(T(x))$$

where $\mathcal{F}$ *is the space of function (observables) invariant under the action of the Koopman operator.*

In essence, the P-F operator governs the evolution of measures (or distributions) under the dynamical map (flow for continuous-time systems), whereas, the Koopman operator governs the evolution of functions under the dynamical map (see Fig. S2) and satisfy the following properties:

Property 7. *Following properties for the Koopman and Perron-Frobenius operators can be stated.*

a). For any function $h \in \mathcal{F}$ *such that* $h \geq 0$*, we have* $[\mathbb{U}h](x) \geq 0$ *and hence Koopman is a positive operator.*

b). If we define P-F operator to act on the space of densities i.e., $L_1(X)$ *and Koopman operator on space of* $L_\infty(X)$ *functions, then it can be shown that the P-F and Koopman operators are dual to each other as follows*

$$\langle \mathbb{U}f, g \rangle = \langle f, \mathbb{P}g \rangle$$

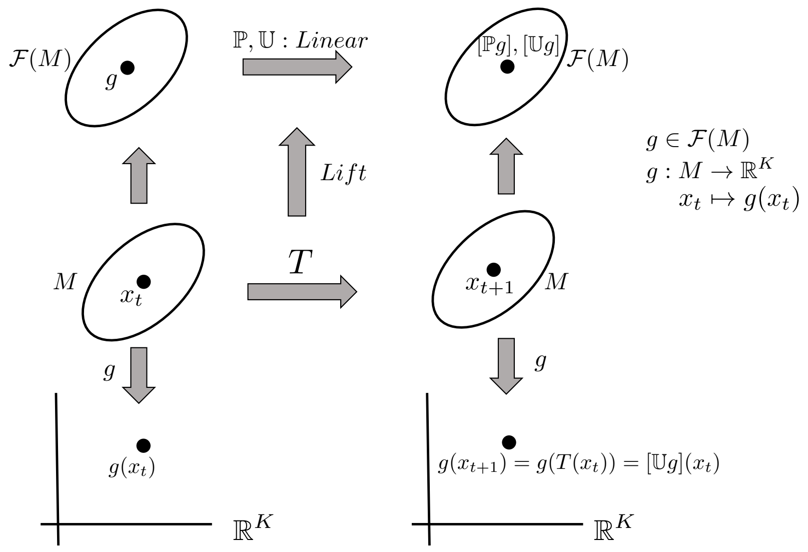

Figure S2: Perron-Frobenius and Koopman operators corresponding to a dynamical system.

where $f \in L_\infty(X)$ *and* $g \in L_1(X)$.

c). For $g(x) \geq 0$, $[\mathbb{P}g](x) \geq 0$.

d). Let $(X, \mathcal{B}, \mu)$ *be the measure space where* μ *is a positive but not necessarily an invariant measure, then the P-F operator satisfies following property.*

$$\int_X [\mathbb{P}g](x) d\mu(x) = \int_X g(x) d\mu(x)$$

Hence, the P-F and the Koopman operators are adjoint to one another and are linear operators even if the underlying system is nonlinear. While the analysis is made tractable by linearity, the trade-off is that these operators are typically infinite-dimensional. In particular, the P-F and Koopman operators lift a dynamical system from a finite-dimensional space to generate an infinite-dimensional linear system on the space of distributions and functions respectively.

Data-driven Computation of Information Transfer

Computation of Finite-dimensional Koopman Operator

In this subsection, we discuss the data-driven approach to compute the information transfer for a linear dynamical system. For details, see [7, 8].

Consider snapshots of the data set obtained from simulating a discrete-time random dynamical system $z \mapsto T(z, \xi)$ or from an experiment

$$\mathcal{Z} = [z_0, z_1, \ldots, z_M] \tag{13}$$

where $z_i \in Z \subset \mathbb{R}^N$. The data set could be corrupted by either norm-bounded process or measurement noise or both. In this case, the existing Dynamic Mode Decomposition (DMD) or Extended Dynamic Mode Decomposition (EDMD) algorithms [9, 10] often generate erroneous Koopman operator and in [11, 12], the

authors provided algorithms, based on robust optimization techniques, that compute the Koopman operator in the presence of noise in the data.

Define a vector-valued function $\mathbf{\Psi} : X \to \mathbb{C}^K$ as

$$\mathbf{\Psi}(z) := \begin{bmatrix} \psi_1(z) & \psi_2(z) & \cdots & \psi_K(z) \end{bmatrix} \tag{14}$$

With this, the optimization problem for computation of the finite-dimensional Robust Koopman operator ($\mathbf{K}$) can be formulated as [11, 12, 13]

$$\min_{\mathbf{K}} \parallel \mathbf{G}\mathbf{K} - \mathbf{A} \parallel_F + \lambda \parallel \mathbf{K} \parallel_F \tag{15}$$

where

$$\begin{aligned} \mathbf{G} &= \frac{1}{M} \sum_{m=1}^{M} \mathbf{\Psi}(z_m)^\top \mathbf{\Psi}(z_m) \\ \mathbf{A} &= \frac{1}{M} \sum_{m=1}^{M} \mathbf{\Psi}(z_m)^\top \mathbf{\Psi}(z_{m+1}). \end{aligned} \tag{16}$$

and λ, the regularization parameter, is dependent on the noise bound. For details, see [11, 12, 13].

Note that, since the Koopman operator is a linear operator on the space of functions, the finite-dimensional Robust Koopman operator is a linear operator (a matrix) on the space of the observables $\mathbf{\Psi}$. Hence, even if the underlying system is nonlinear, the Koopman form is always linear on the space of the observables and hence we have a linear representation of the underlying system. However, it is to be noted that the Koopman representation is not equivalent to linearization around some point in the state space, but is an exact linear representation of the underlying system in the space of functions. Thus, unlike linearization, which is valid locally, the Koopman model is a linear representation which is valid globally on the state space. Intuitively, Koopman operator can be thought of as a nonlinear change of co-ordinates which changes the nonlinear evolution of a dynamical system defined on Euclidean space to linear evolution on function space.

Computation of Information Transfer

For computing the information transfer measure, we need to compute two different Koopman operators, one corresponding to the original dynamics and one corresponding to the frozen dynamics and use these operators to compute to information transfer. The algorithm is shown in Fig. S3.

For computing the information transfer measure, we use $\mathbf{\Psi}(z) = z$, so that the robust Koopman operator obtained from the optimization problem (15) gives the system matrix. Let $\bar{A} = \mathbf{K} \in \mathbb{R}^{N \times N}$ be the estimated system dynamics obtained using optimization formulation (15). Under the assumption that the initial covariance matrix is $\bar{\Sigma}(0)$, the conditional entropy $H(y_{t+1}|y_t)$ for the non-freeze case is computed using the following formula [1, 2].

$$H(y_{t+1}|y_t) = \frac{1}{2} \log |\bar{A}_{yx} \bar{\Sigma}_y^S(t) \bar{A}_{yx}^\top + \left(\frac{\lambda}{3}\right)^2 I|. \tag{17}$$

where $|\cdot|$ is the determinant, λ is the bound on the process noise, $\bar{\Sigma}(t)$ evolves according to the equation $\bar{\Sigma}(t) = \bar{A}\bar{\Sigma}(t-1)\bar{A}^\top + \sigma^2 I$ and $\bar{\Sigma}_y^S(t)$ is the Schur complement of y in the covariance matrix $\bar{\Sigma}(t)$. In computing the entropy, we assume that the noise is i.i.d. Gaussian with covariance $\Sigma = \text{diag}(\sigma^2, \cdots, \sigma^2)$ so that one can take the bound as $\lambda = 3\sigma$, to cover the essential support of the Gaussian distribution.

The computation of entropy for the frozen dynamics is a bit tricky, and for simplicity, we describe the procedure for a two-state system. The method generalizes easily for the general N-dimensional systems. Let the obtained time series data be given by

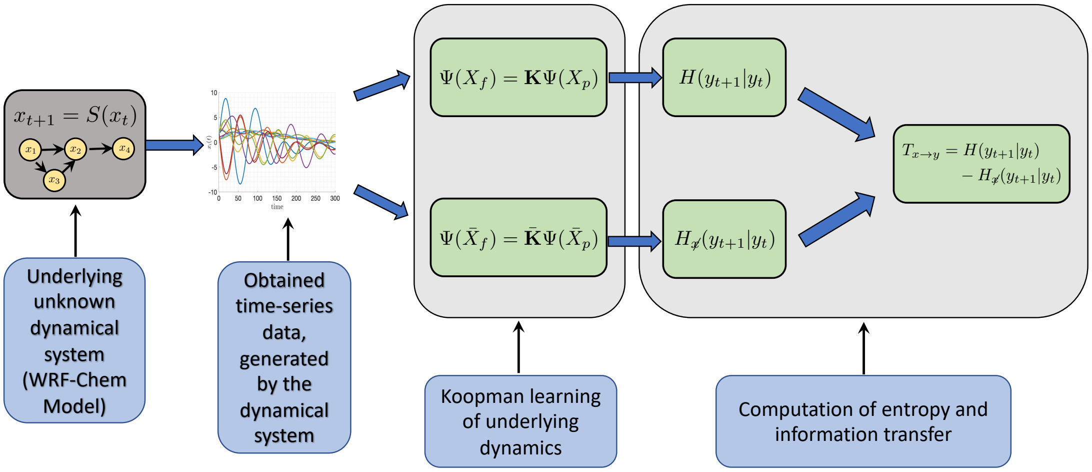

Figure S3: **Graphical representation of the algorithm for computation of information transfer from time-series data:** We have an unknown dynamical system from which time-series measurements $[x_1, \cdots, x_M] \in \mathbb{R}^{n\times M}$ of the states are obtained. The obtained time-series data is lifted to a space of functions using the Koopman observables $\Psi(x)$ such that $\Psi(X_p) = [\Psi(x_1), \cdots, \Psi(x_{M-1})]$ and $\Psi(X_f) = [\Psi(x_2), \cdots, \Psi(x_M)]$ and the linear Koopman operator ($\mathbf{K}$) in this space is obtained as a solution to a least squares problem. The Koopman operator for the frozen dynamics $\bar{\mathbf{K}}$ is computed using the frozen data (Eq. (19)). Thus, Koopman learning identifies two linear models [6, 14], namely the underlying original dynamical system and the frozen dynamical system, and these linear models are used to compute the relevant conditional entropies which in turn are used to compute the information transfer measure.

$$\mathcal{D} = \left[\begin{pmatrix} x_0 \\ y_0 \end{pmatrix}, \begin{pmatrix} x_1 \\ y_1 \end{pmatrix}, \dots, \begin{pmatrix} x_{M-1} \\ y_{M-1} \end{pmatrix} \right] \tag{18}$$

To emulate the effect of freezing of x dynamics (4) we modify the original data set (18) as follows.

$$\mathcal{D}_{\not{x}} = \left[\left\{ \begin{pmatrix} x_0 \\ y_0 \end{pmatrix}, \begin{pmatrix} x_0 \\ y_1 \end{pmatrix} \right\}, \left\{ \begin{pmatrix} x_1 \\ y_1 \end{pmatrix}, \begin{pmatrix} x_1 \\ y_2 \end{pmatrix} \right\}, \dots, \left\{ \begin{pmatrix} x_{M-1} \\ y_{M-1} \end{pmatrix}, \begin{pmatrix} x_{M-1} \\ y_M \end{pmatrix} \right\} \right] \tag{19}$$

Then the Koopman operator on this modified data set identifies the optimal linear mapping that propagates the state $[x_{t-1} \quad y_{t-1}]^\top$ to $[x_{t-1} \quad y_t]^\top$ (i.e., x is frozen) for $t = 1, 2, \dots, M$. Thus, the Koopman operator for this modified data set estimates the frozen dynamics $\bar{A}_{\not{x}}$. Once the frozen model is calculated, the entropy $H_{\not{x}}(y_{t+1}|y_t)$ is calculated using exactly the same procedure outlined for computing $H(y_{t+1}|y_t)$, but this time applied to $\bar{A}_{\not{x}}$. Finally, the information transfer from $x \to y$ is computed using the formula

$$T_{x\to y} = H(y_{t+1}|y_t) - H_{\not{x}}(y_{t+1}|y_t).$$

The algorithm for computing the information transfer from time-series data can be summarized as follows:

Algorithm 1 Information Transfer: Linear System

---

1. From the original data set (18), compute the estimate of the system matrix $\bar{A}$ using the optimization formulation (15).
2. Assume $\bar{\Sigma}(0)$ and compute $\bar{\Sigma}(t)$ using Eq. (11). Determine $\bar{A}_{yx}$ and $\bar{\Sigma}_y^S$ to calculate the conditional entropy $H(y_{t+1}|y_t)$ using (17).
3. From the original data set (18) form the modified data set for the x freeze dynamics as given by Eq. (19).
4. Follow steps (1)-(2) to compute the conditional entropy $H_{\not{x}}(y_{t+1}|y_t)$.
5. Compute the transfer $T_{x\to y}$ as $T_{x\to y} = H(y_{t+1}|y_t) - H_{\not{x}}(y_{t+1}|y_t)$.

Causal Structure of Chemical Processes

A chemical process can be thought of as a dynamical system [15, 16, 17] with the equations of motion being given by the rate kinetics equation. Here we consider two different chemical reactions to show how the information transfer measure can be used to discover the causal structure of a chemical process. These examples form the basis of our study of the WRF-Chem data.

A linear equation

Consider the chemical reaction

$$CaCO_3 \to CaO + O_2. \tag{20}$$

From the dynamic approach of the law of mass action, the differential equations governing the concentrations of the different chemical species are

$$\begin{aligned} \frac{d[CaCO_3]}{dt} &= -k[CaCO_3] \\ \frac{d[CaO]}{dt} &= k[CaCO_3] \\ \frac{d[O_2]}{dt} &= k[CaCO_3], \end{aligned} \tag{21}$$

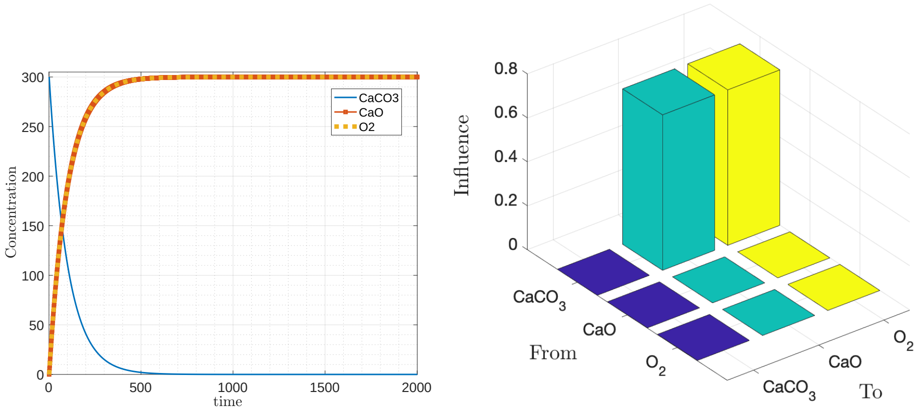

(a)

(b)

Figure S4: (a) Evolution of the concentration of the reactants and the products for the reaction 20. (b) Information transfer between the different chemical species for the reaction 20.

where $[\cdot]$ denotes the concentration of any species at time t and k is the rate constant.

For simulation purposes, the initial concentration of $CaCO_3$ was chosen to be 3×10^2 and that of the products was assumed to be zero. For simplicity, we considered only the forward reaction and set the rate constant to unity. The time evolution of the concentrations of the chemicals is shown in Fig. S4(a).

The information transfer between the different chemicals (their concentrations, in particular), computed from the time-series data of the concentrations of each species, is shown in Fig. S4(b). It can be seen that the information transfer from the reactant ($CaCO_3$) to both the products (CaO and O_2) is non-zero, whereas the information flow from both the products to the reactant is zero. This is concurrent with the intuition that the products cannot influence the reactants, whereas it is the reactants that influence the products.

A chemical process can be thought of as a dynamical system [15, 16, 17] with the equations of motion being given by the rate kinetics equation. Here we consider two different chemical reactions to show how the information transfer measure can be used to discover the causal structure of a chemical process. These examples form the basis of our study of the WRF-Chem data.

A nonlinear equation

Consider the chemical reaction

$$C_3H_8 + 5O_2 \rightarrow 4H_2O + 3CO_2, \tag{22}$$

such that the differential equations for the evolution of the different concentrations are

$$\begin{aligned} \frac{d[C_3H_8]}{dt} &= -k[C_3H_8][O_2]^5, & \frac{d[O_2]}{dt} &= -k[C_3H_8][O_2]^5 \\ \frac{d[H_2O]}{dt} &= 4k[C_3H_8][O_2]^5, & \frac{d[CO_2]}{dt} &= 3k[C_3H_8][O_2]^5, \end{aligned} \tag{23}$$

with k being the rate constant, which was set to unity for generating time series data. The initial concentrations of both the reactants were chosen to be 3×10^2 and those of the products were chosen to be zero.

The time evolution of the concentrations for the first 20 times steps is shown in Fig. S5(a). For the computation of information transfer between the chemical species, data were obtained for 200 time steps and the information transfer between the various species is shown in Fig. S5(b). In this example, where the governing equations are highly nonlinear, it can be seen that the reactants have substantial influence on the products, whereas the influence of the products on the reactants is minimal. However, it is not exactly zero,

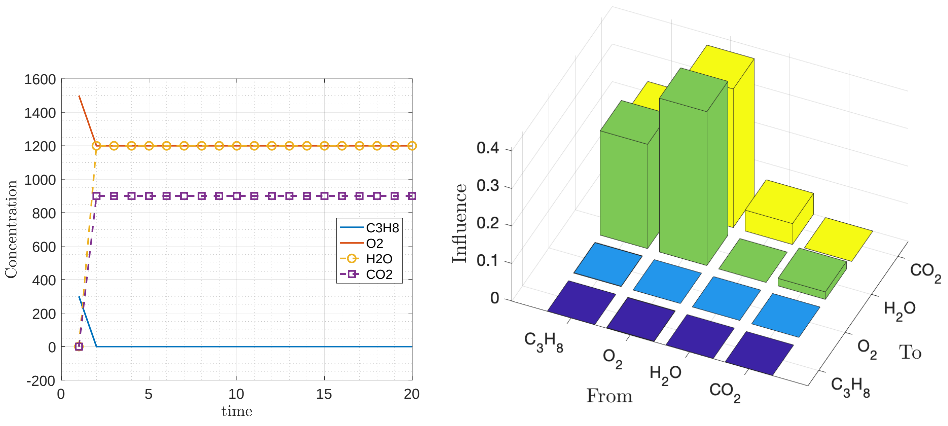

(a) (b)

Figure S5: (a) Evolution of the concentration of the reactants and the products for the chemical reaction 22. (b) Information transfer between the different chemical species for the chemical reaction 22.

and this is because of the following. In real experiments, the concentrations are measured using sensors, and it is natural that there is some noise in the measurements. To simulate the effect of noise, we added independent and identically distributed Gaussian noise of zero mean and unit variance to the concentration data and computed the influence with the noisy data. It is the uncertainty in the data that results in a small influence of the products on the reactants, but still, it is small, and one can set a lower limit (≈ 0.1) and set any information transfer value below 0.1 to zero. This is a standard procedure in data-driven methods, and with it, one can identify the cause and effects of a chemical process.

Effect of Noisy Data and Extraneous Variables on Causal Structure of WRF-Chem Model

Effect of Noise

To demonstrate the effects of noise in our IEPOX-SOA analyses, we performed new sets of simulations where we added measurement noise to the data obtained from the WRF-Chem model. At each time point, we added independent and identically distributed (i.i.d.) Gaussian noise with zero mean and variance of 0.2 to the normalized data and looked at the influence of the different features on total tetrol particle (Note: total terol particles are dominant components of IEPOX-SOA) concentrations near the surface i.e. at 0-500m altitudes. As for choosing the value of the variance, typically the variance of measurement noise can be assumed to be 5% $\sim$ 15% [18], and in our analysis we chose the variance as 0.2 (which corresponds to 20%) and showed that the most influential variables are correctly identified with this high measurement noise. The results are shown in Fig. S6.

We find that in both cases (noise-free and noisy data), the information transfer algorithm identifies SO_4 and particle water as the most influential variables affecting tetrol particles. However, the relative IT from other less influential variables to tetrol particles changes when noise is added to the same dataset.

Presence of Extraneous Variables

As discussed in the main text, the presence of unknown extraneous variables can lead to the *discovery* of causal links which are not there in the system. But if one has only the time series data of some states (say m) of a system and he/she has no other knowledge about the actual system (he/she does not even know that the actual dimension of the system is n, with $n > m$), then no matter what algorithm is used to discover the causal structure it just won't capture the true causal structure.

To this end, we performed additional causal analyses with more extraneous variables (chemical and transport related variables) derived from WRF-Chem outputs using 18 input variables as opposed to 11 input variables. The results are shown in Fig. S7. It can be seen that for both 18 and 11 input variable

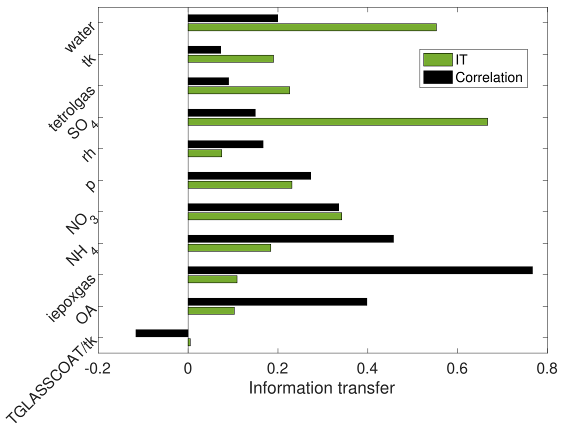

(a)

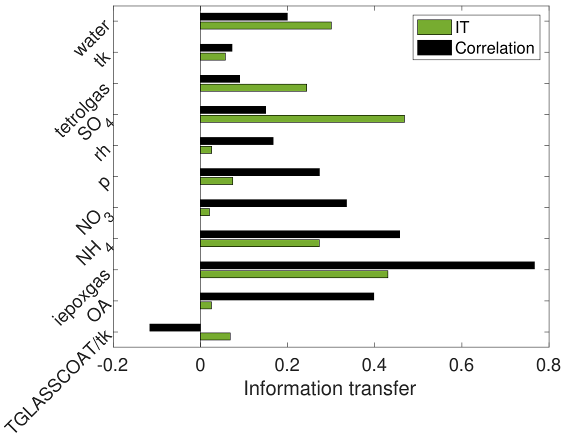

(b)

Figure S6: (a) Information transfer to total tetrol and correlation of total tetrol with other variables for altitude 0-500m for noise-free data. (b) Information transfer to total tetrol and correlation of total tetrol with other variables for altitude 0-500m with i.i.d. Gaussian measurement noise.

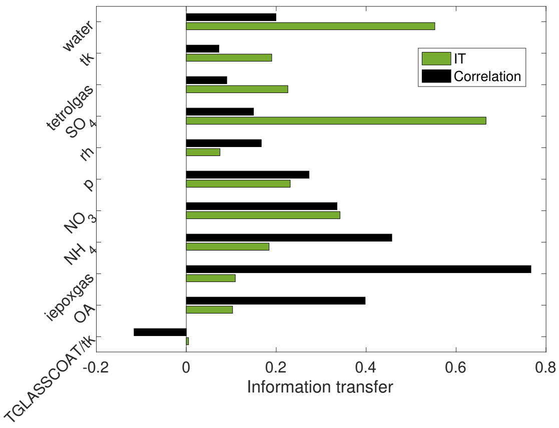

(a)

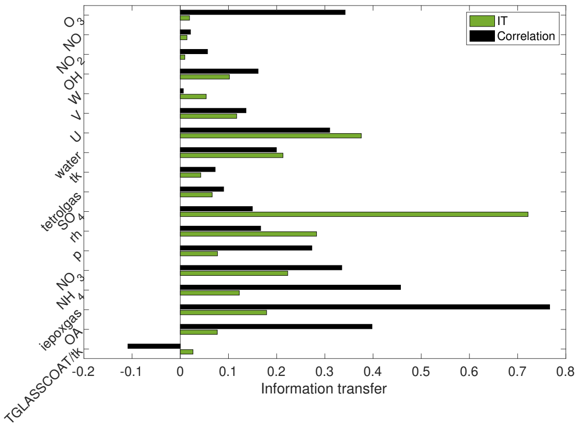

(b)

Figure S7: (a) Information transfer to total tetrol and correlation of total tetrol with other variables for altitude 0-500m with 11 input variables. (b) Information transfer to total tetrol and correlation of total tetrol with other variables for altitude 0-500m with 18 input variables.

scenarios, our information transfer algorithm identifies SO_4 as the most influential variable affecting total tetrol particles. However, when 18 variables are considered, the horizontal and vertical velocities (U, V, W) also show up as influential variables, which is consistent with the expectation that total tetrol particles are affected by transport and mixing, in addition to chemistry.

In summary, it is encouraging that adding extraneous variables related to chemistry (O_3, NO, NO_2, OH radicals) and horizontal velocity (U,V) and vertical velocity W to the existing set of 11 input variables does not change the dominant feature (SO_4) affecting total tetrol particles near the surface. However, the IT identifies additional variables i.e. horizontal and vertical velocity components affecting transport and mixing of IEPOX-SOA near the surface.

References

[1] S. Sinha, U. Vaidya, Causality preserving information transfer measure for control dynamical system, in: 2016 IEEE 55th Conference on Decision and Control (CDC), IEEE, 2016, pp. 7329–7334.

[2] S. Sinha, U. Vaidya, On information transfer in discrete dynamical systems, in: 2017 Indian Control Conference (ICC), IEEE, 2017, pp. 303–308.

[3] C. E. Shannon, A mathematical theory of communication, The Bell system technical journal 27 (3) (1948) 379–423.

[4] L. Boltzmann, Über die mechanische Bedeutung des zweiten Hauptsatzes der Wärmetheorie:(vorgelegt in der Sitzung am 8. Februar 1866), Staatsdruckerei, 1866.

[5] E. T. Jaynes, Gibbs vs boltzmann entropies, American Journal of Physics 33 (5) (1965) 391–398.

[6] A. Lasota, M. C. Mackey, Chaos, Fractals, and Noise: Stochastic Aspects of Dynamics, Springer-Verlag, New York, 1994.

[7] S. Sinha, U. Vaidya, Data-driven approach for inferencing causality and network topology, in: 2018 Annual American Control Conference (ACC), IEEE, 2018, pp. 436–441.

[8] S. Sinha, U. Vaidya, On data-driven computation of information transfer for causal inference in discrete-time dynamical systems, Journal of Nonlinear Science 30 (4) (2020) 1651–1676.

[9] P. J. Schmid, Dynamic mode decomposition of numerical and experimental data, Journal of Fluid Mechanics 656 (2010) 5–28.

[10] M. O. Williams, I. G. Kevrekidis, C. W. Rowley, A data–driven approximation of the koopman operator: Extending dynamic mode decomposition, Journal of Nonlinear Science 25 (6) (2015) 1307–1346.

[11] S. Sinha, B. Huang, U. Vaidya, Robust approximation of koopman operator and prediction in random dynamical systems, in: 2018 Annual American Control Conference (ACC), IEEE, 2018, pp. 5491–5496.

[12] S. Sinha, B. Huang, U. Vaidya, On robust computation of koopman operator and prediction in random dynamical systems, Journal of Nonlinear Science 30 (5) (2020) 2057–2090.

[13] S. Sinha, S. P. Nandanoori, D. Barajas-Solano, Online real-time learning of dynamical systems from noisy streaming data, Nature Scientific Reports 13, 22564 (2023) 1–15.

[14] M. Budisic, R. Mohr, I. Mezic, Applied koopmanism, Chaos 22 (2012) 047510–32.

[15] G. F. Oster, A. S. Perelson, A. Katchalsky, Network thermodynamics: dynamic modelling of biophysical systems, Quarterly reviews of Biophysics 6 (1) (1973) 1–134.

[16] S. A. Frank, Input-output relations in biological systems: measurement, information and the hill equation, Biology direct 8 (1) (2013) 1–25.

[17] A. van der Schaft, B. Maschke, A port-hamiltonian formulation of open chemical reaction networks, in: Advances in the Theory of Control, Signals and Systems with Physical Modeling, Springer, 2010, pp. 339–348.

[18] M. J. Grimble, Robust industrial control systems: optimal design approach for polynomial systems, John Wiley & Sons, 2006.
